# Rapid mortality transition of Pacific Islands in the 19th century

**DOI:** 10.1017/S0950268816001989

**Published:** 2016-09-09

**Authors:** B. S. PENMAN, S. GUPTA, G. D. SHANKS

**Affiliations:** 1 Department of Zoology, University of Oxford, Oxford, UK; 2 Australian Army Malaria Institute, Enoggera, Australia; 3 University of Queensland, School of Public Health, Brisbane, Australia

**Keywords:** History of epidemics, infectious disease, mass mortality, Pacific islands

## Abstract

The depopulation of Pacific islands during the 16th to 19th centuries is a striking example of historical mass mortality due to infectious disease. Pacific Island populations have not been subject to such cataclysmic infectious disease mortality since. Here we explore the processes which could have given rise to this shift in infectious disease mortality patterns. We show, using mathematical models, that the population dynamics exhibited by Pacific Island populations are unlikely to be the result of Darwinian evolution. We propose that extreme mortality during first-contact epidemics is a function of epidemiological isolation, not a lack of previous selection. If, as pathogens become established in populations, extreme mortality is rapidly suppressed by herd immunity, Pacific Island population mortality patterns can be explained with no need to invoke genetic change. We discuss the mechanisms by which this could occur, including (i) a link between the proportion of the population transmitting infectious agents and case-fatality rates, and (ii) the course of infection with pathogens such as measles and smallpox being more severe in adults than in children. Overall, we consider the present-day risk of mass mortality from newly emerging infectious diseases is unlikely to be greater on Pacific islands than in other geographical areas.

## INTRODUCTION

Pacific islands were the last places on earth that humans reached as Melanesians expanded from New Guinea, and Polynesians voyaged in ocean-going canoes to more distant and isolated islands [1]. Over millennia this resulted in great genetic diversity between islands but a large degree of genetic homogeneity on any particular island due to founder effects [2]. The Pacific islands were also the last area contacted by Western explorers on missions of discovery and colonization. Soon after first contact of island societies with distant populations was established, lethal infectious disease epidemics began [3]. Despite the outsiders often having no obvious illness, epidemics of poorly characterized respiratory and gastrointestinal infections devastated isolated Pacific Island populations, severely dislocating and at times destroying their social systems [4]. These events pre-dated the understanding of microbes as the cause of infectious diseases such that the collapse of indigenous island populations was often interpreted in racial terms. Yet even after the development of germ theory, notions of white superiority persisted into the 20th century: ‘Only a race which has undergone evolution against the diseases of crowds is capable of civilization’ [5]. This thinking became even more evident when the 1918 influenza pandemic caused great differential mortality of co-located groups with up to a fifth of Samoans dying while New Zealand soldiers were relatively spared mortality but not infection [6].

Today Pacific Islanders have reversed the colonization process to the United States, Australia and New Zealand with large growing populations that only show traces of increased infectious disease susceptibility [7]. Highly vulnerable Pacific populations underwent a rapid transition during the first few generations following entry into the global pathogen pool such that their current distinctive health problems (e.g. adult-onset diabetes) are not of an infectious nature. These changes largely occurred during the 19th century before accurate enumeration arrived in Pacific islands [3].

**Fig. 1.**
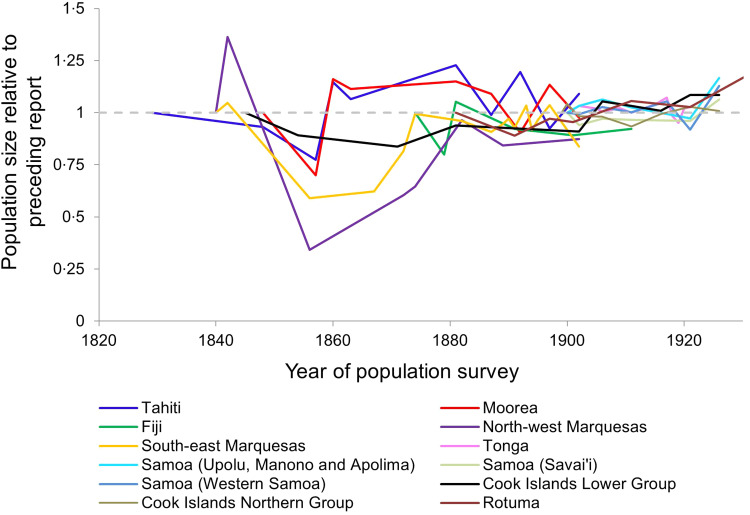
Population dynamics in Pacific Island populations, 1830–1930. Here we visualize data from census reports for various indigenous populations of Pacific islands. All reports other than those for Rotuma are as compiled and reviewed by Norma McArthur [3]; the Rotuma data came from other reports including original documents transcribed in the 1960s by Dr Alan Howard [20, 46]. To facilitate comparison between populations of different sizes, we display each report of population numbers relative to the size of the population at the preceding report. Each population's time series begins with a value of 1, at the first time point for which we have a report. Values >1 indicate that the population grew since the previous report; values <1 indicate that the population shrank since the previous report. The first available Fiji datapoint comes from 1879, but Fiji was estimated to have lost >1/5 of the indigenous Fijian population over a 4-month period in 1875 due to a measles outbreak [3], so for the Fiji time series we extrapolated a higher starting population in 1875.

Figure 1 summarizes the time-series data that are available from census records between 1830 and 1930 [3]. Certain features are attributable to known outbreaks of infectious disease, for example a devastating measles outbreak in Fiji in 1875, and population losses in Western Samoa as a consequence of the 1918 influenza pandemic [6, 8]. Most of the populations for which data are available prior to 1860 – specifically Tahiti and Moorea and the South-east and North-west Marquesas – display a strikingly similar pattern: a single event involving an initial loss of between 20% and 70% of the population, followed by a slow period of recovery, in which no similar crashes occur. Here we seek to understand the evolutionary and epidemiological processes underlying such dynamics.

We first briefly review the Pacific islands' early experience of epidemics caused by specific infectious agents:

*Dysentery*. Dysentery was often the first large lethal epidemic said to have occurred on many Pacific islands. In 1804 such an epidemic in Hawaii is said to have killed so many (estimate range 5000-15 000) that the living were unable to bury the dead [9]. Despite the description ‘sour starch water flowed frequently from the bowels’ it seems unlikely that cholera arrived in Hawaii prior to the first global pandemic and a more likely diagnosis would be shigellosis spread from ships’ crews [10]. Shigellosis causing blood and pus in stools was a common problem on contract labour ships (*black-birders*) as described in 1890 arriving in Fiji from Solomon Islands [11, 12]. Half of those infected died within 4–10 days with post-mortem examinations showing an acutely inflamed large bowel with black-green deposits. The presence of genitourinary ulcers as well severe arthritis in some survivors indicates a post-dysentery syndrome caused by *Shigella* was likely [11, 13]. Dysentery was such a serious problem in sugar plantations on Fiji that a special investigation was commissioned in 1910. Prior to the antibiotic era, fatal cases were marked by inflammation throughout the small and large intestine with ‘frog-spawn stools’ [14].

*Measles*. Measles could be diagnosed from its distinctive skin rash and is known to have caused major lethal epidemics when first introduced in Hawaii, Fiji, Tonga, Samoa and Rotuma [8, 15–18]. Mortality of up to a quarter of the entire population occurred across all ages including previously healthy young adults. Measles was particularly dangerous on isolated islands because a large proportion of the adult population were simultaneously ill leaving few to care for the sick [19]. Severe forms of measles particularly with sub-acute inflammatory gastrointestinal symptoms were common on Pacific islands [20]. Black or haemorrhagic measles was particularly lethal. Sequential measles epidemics occurred in Fiji with progressively smaller case-fatality rates [21]. High-lethality measles epidemics ceased once the most isolated Pacific islands were incorporated into the global system of air travel [22].

*Influenza*. Epidemics of respiratory disease often followed the arrival of ships to isolated Pacific islands. The clinical diagnosis of influenza virus infection is problematic but the association of lethal outbreaks on Pacific islands with influenza pandemics of 1890 and 1918 indicates the likelihood that influenza was the actual infectious agent involved [23–25]. Severe forms of influenza with secondary bacterial pneumonia were particularly apparent during the 1918–1920 pandemic, and contributed to the large mortality differences between immigrants and indigenous Pacific peoples on Fiji, New Zealand, Nauru, Samoa, Saipan and Hawaii [6, 10, 24, 26]. In islands bypassed by the 1918–1920 pandemic such as Niue, New Guinea and Marshall Islands, deadly influenza epidemics occurred decades later even when the viruses involved were not particularly lethal in other countries [27–30]. Polynesian or Micronesian ethnicity was not an essential characteristic as seen during influenza epidemics on isolated Aleutian islands near Alaska well into the 20th century [31]. During the 2009 influenza pandemic mortality on Pacific islands was not remarkably different from other countries globally [32].

*Smallpox*. Because of smallpox's extraordinary lethality and its distinctive pustular rash, quarantine efforts were largely able to limit its ravages within the Pacific to Hawaii, Guam, Caroline Islands, French Polynesia and Papua New Guinea [33]. Devastating smallpox epidemics swept the northern coast of New Guinea in 1872, 1893 and 1895 during the German colonial establishment of a plantation economy. In 1854, 2000 of 5000 persons died on the Caroline Islands from smallpox and approximately one-third of the population of Guam perished in 1856 [33]. Probably the Pacific group most affected by smallpox were Australian Aboriginal peoples. Epidemics in the 1780s, 1829–1831 and the 1860s, likely originating from fisherman arriving in remote areas of the Northern Territory, killed an uncounted but large proportion of Australia's indigenous inhabitants [34]. Severe and universally fatal forms of smallpox especially haemorrhagic smallpox appear to fit some of the descriptions of these epidemics [35].

In an attempt to better understand the Pacific peoples’ rapid transition from crisis infectious disease mortality to current infection mortality patterns we have devised a mathematical model. In our model, three hypothetical pathogens arrive in an immunologically naive population. Resistance alleles exist to each of these pathogens, and the model can simulate their possible change in frequency. Our model also allows us to incorporate a herd immunity mortality feedback, whereby the higher the level of herd immunity to a pathogen, the lower the case-fatality rate of that pathogen. We identify sets of pathogen-specific mortality rates and population starting frequencies of protective alleles that are consistent with the 19th century mortality transition of Pacific Islanders. We show that it is unnecessary to invoke changes in allele frequencies (i.e. Darwinian evolution) to explain observed mortality patterns, and that the most plausible pathogen mortality scenarios occur in the presence of herd immunity mortality feedbacks.

## METHODS

### Summary of approach

We considered the arrival of three new pathogens in a naive population. We allowed there to be three host loci, independently determining disease resistance to the three pathogens, with a wild type and a resistant allele at each. For each of the 27 possible host genotypes in the population, we used a standard compartmental epidemiological model (consisting of linked ordinary differential equations) to simulate the rate of change of numbers of susceptible, infected and recovered individuals [see equations (1)–(10) at the end of the Methods section].

We wished to compare and contrast a genetic explanation for the Pacific Island population mortality pattern with an epidemiological one. To allow for Darwinian evolution, we linked the birth rates of each host genotype in the model to the frequencies of the relevant alleles in the surviving population, so that the relative numbers of individuals of different genotypes could change in response to selection from the infectious disease agent. To capture possible epidemiological mechanisms, we allowed infectious disease mortality to be a function of the proportion of the population already immune: a herd immunity feedback. The strength of the herd immunity feedback was determined by parameter *γ* [see also equations (1)–(10) at the end of the Methods section]. If *γ* is non-zero, the more individuals there are who are immune to a specific pathogen, the lower the mortality rate for any given infection with that pathogen. This could occur if (i) an infection has more severe effects in adults than in children (the higher the proportion of immune individuals, the lower the age of first infection), or (ii) there is a link between rate of exposure and mortality, and the more infected individuals there are at a particular point in time, the higher the exposure to the pathogen. Both of these phenomena are explored in more detail in the Discussion section.

### Analysing the behaviour of the model

**Fig. 2.**
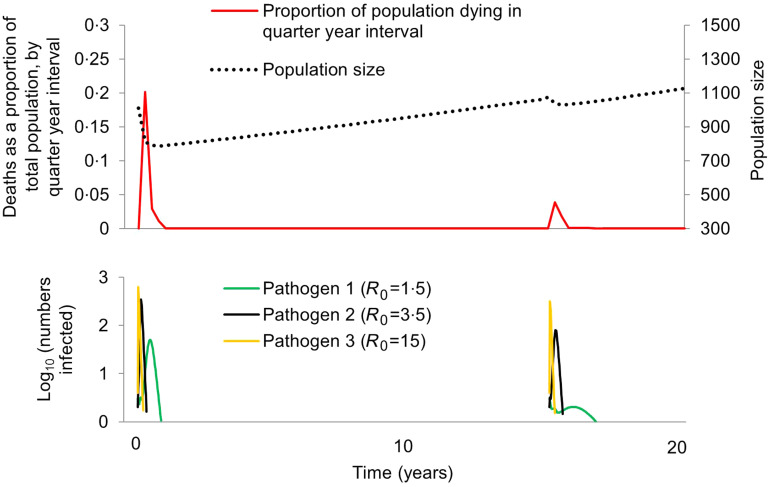
Pacific Island population first-contact-like dynamics within the model. Here we illustrate a scenario in which the first introduction of three pathogens leads to a ⩾20% loss in population size over a quarter year period, but no more than a 5% loss over a quarter year period when the pathogens are introduced for a second time. Parameter values were as follows: *θ*_1_ = 0·1, *θ*_2_ = 0·35 and *θ*_3_ = 0·01, and *γ* = 1. All other parameters are given in the Methods section. No protective alleles were included in this scenario. Population size is indicated by a dotted black line; deaths as a proportion of total population is indicated by a solid red line; numbers infected with each pathogen are indicated by different coloured lines as shown in the legend.

We considered scenarios in which the maximum proportion of the population to die in a quarter-year interval was ⩾20% and <70% between years 0 and 5, but ⩽5% between years 15 and 20, to have captured dynamics consistent with Pacific Island population first-contact epidemics (Fig. 2). Critical features of these dynamics are: (i) population loss occurs over a short time – consistent with the reports noted in the introduction of epidemics in which so many were affected that ‘the living could not bury the dead’, (ii) within decades, the population was no longer subject to such devastating losses – consistent with the population recovery pattern exhibited by Tahiti and Moorea (Fig. 1). We used Latin Hypercube Sampling (specifically the LHSdesign function of Matlab v. R2014b) to ensure a fair sampling of parameter space and explore which combinations of either (i) starting frequencies of protective alleles or (ii) pathogen-specific mortality rates were capable of generating these first-contact-like dynamics, in the presence or absence of a herd immunity feedback effect.

### Parameter values and assumptions

In order to make this modelling exercise feasible, certain parameter values had to be fixed. We set the recovery rate of each pathogen such that the average duration of infectiousness was 1·5 weeks (*σ*_*x*_ = 52/1·5, see end of Methods section for equations which completely describe our model). Of the types of pathogens which may have caused Pacific Island population mortality, the duration of infectiousness for measles is ~8 days, that of influenza, 7–14 days and that of smallpox ⩾7 days [source: World Health Organisation disease factsheets and frequently asked questions (http://www.who.int/en/)]. A diarrhoeal disease such as dysentery could be infectious for between 3 days and up to 4 weeks depending on the duration of symptoms. Transmission parameters were given the following values *β*_1_ = 52, *β*_2_ = 121·3 and *β*_3_ = 520, making the basic reproductive numbers of each pathogen: *R*_0(1)_ = 1·5, *R*_0(2)_ = 3·5 and *R*_0(3)_ = 15. These values were chosen to lie within the plausible range of values of *R*_0_ for pathogens that could have arrived in Pacific islands during the 19th century, and also to contrast low and high *R*_0_ pathogens as potential culprits for the bulk of the mass mortality.

At the beginning of each simulation there were 1000 susceptible individuals in the population, intended to simulate a small island community. Individuals carrying no protective alleles and infected with pathogen 1 (*n* = 3), pathogen 2 (*n* = 2) or pathogen 3 (*n* = 4) were added to the population at the beginning of every simulation (time = 0), then three, two and four extra individuals, infected with pathogens 1, 2 and 3 respectively, and with no protective alleles, were added at time = 15 years. This reintroduction allowed for the possibility that the pathogens died out after their initial introduction: a likely occurrence in a small population where susceptible individuals are quickly exhausted. The growth rate of the population (*r*) was fixed at 5%, and the average lifespan of the host population in the absence of the newly arrived infections was 35 years (*μ* = 0·029). These parameters were intended to be plausible for 19th century human populations. We must stress that the exact values of these parameters are relatively unimportant. We seek to understand the broad phenomenon of a large population crash followed by no further such crashes, not to claim that we have precisely represented the dynamics of a specific island, for which exact estimates of lifespans in the absence of infectious diseases are impossible to obtain.

Our model makes two additional assumptions: co-infection is rare enough that we can assume it does not occur (this seems reasonable for pathogens which follow an acute course), and immunity is lifelong. This latter assumption is entirely reasonable for measles and smallpox, but a simplification for influenza and dysentery.

### The complete model

Code to implement the model described in equations (1)–(10) was written in Matlab and executed in Matlab v. R2014b. As noted above, we consider the arrival of three new pathogens in a naive human population. This population has growth rate *r* and, in the absence of infection, individuals die at rate *μ*. There are three potential disease resistance loci in the host, with two alleles at each. There are therefore three possible genotypes at each locus: 1, homozygous for the wild-type allele; 2, heterozygous for a protective allele or 3, homozygous for a protective allele. Each host genotype can be described by a 3-digit identifier (*ijk*). The rate of change of susceptible individuals of genotype *ijk* is given by equation (1):
1
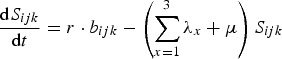


The rate of change of genotype *ijk* individuals with no prior immunity, but infected with pathogen *x* is given by equation (2):
2
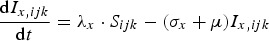


The rate of change of genotype *ijk* individuals immune to pathogen *x* only is given by equation (3):
3
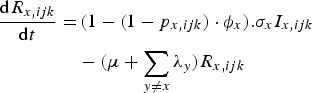


The rate of change of genotype *ijk* individuals, immune to pathogen *x* only and infected with pathogen *y* is given by equation (4):
4
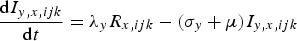


The rate of change of genotype *ijk* individuals, immune to pathogens *x* and *y* only is given by equation (5):
5
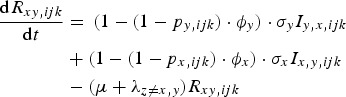


The rate of change of genotype *ijk* individuals, immune to pathogens *x* and *y* and infected with pathogen *z* is given by equation (6):
6
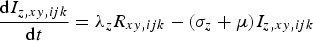


The rate of change of genotype *ijk* individuals, immune to all three pathogens is given by equation (7):
7
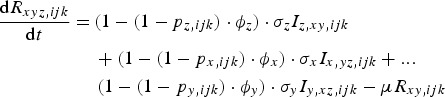


The total number of individuals with genotype *ijk* is given by equation (8):
8
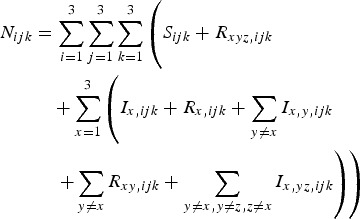


Allele frequencies at the three loci are determined from the values of *N*_*ijk*_, and the term *b*_*ijk*_ allocates different numbers of the new births in the population to different genotypes in Hardy–Weinberg proportions.

Infections with pathogen *x* occur at rate *λ*_*x*_ [equation (9)]:
9
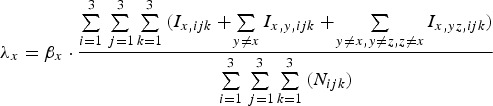

where *β*_*x*_ scales the transmission potential of the pathogen.

A proportion, (1 – *p*_*x,ijk*_)*φ*_*x*_, of individuals infected with pathogen *x* die. Mortality rate *φ*_*x*_ is a function of the total proportion of the population immune to that pathogen, i.e.
10
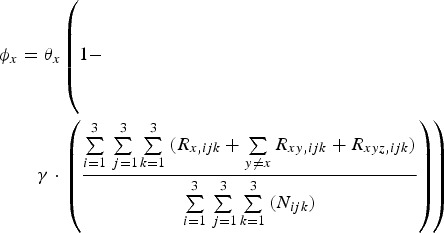
where *θ*_*x*_ is the maximum rate at which pathogen *x* kills and *γ* determines the impact of there already being immune individuals in the population (herd immunity feedback).

*p*_*x,ijk*_ varies the protection against death from infection afforded to individuals on a genotype-specific basis. Upon recovery at rate *σ*_*x*_, individuals are immune to further infection with pathogen *x* for the rest of their lives.

## RESULTS

### First-contact-like dynamics can be achieved in the absence of Darwinian evolution, provided the pathogens involved have highly specific properties

**Fig. 3.**
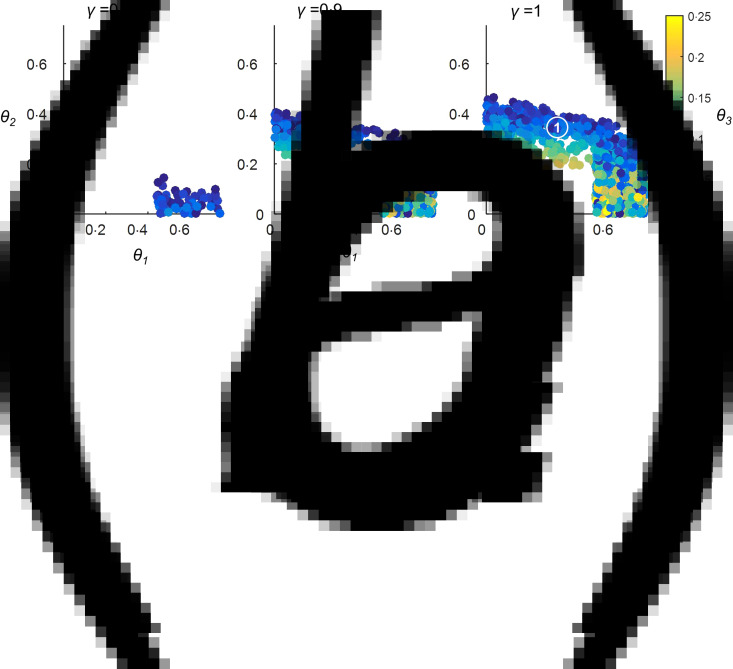
Parameter combinations giving rise to a Pacific Island population first-contact-like pattern, in the absence of genetic changes but including a herd immunity effect on pathogen mortality. No protective alleles were included in this scenario. The maximum mortality rates for each of the three pathogens (*θ*_1−3_) were allowed to vary between 0 and 0·75. Latin Hypercube Sampling was used to identify combinations of *θ*_1−3_ which gave rise to a first-contact-like pattern as described in the Methods section. 25 000 different combinations of values for *θ*_1−3_ were tested. Panels (*a*–*c*) illustrate results for different levels of parameter *γ*, which controlled the extent to which the proportion of the population already immune to a pathogen affected that pathogen's mortality rate. All other parameters were as detailed in the Methods section. Markers 1 and 2 in panel (*c*) indicate two key outcomes that only become possible when *γ* takes values approaching 1. The implications of these outcomes for the types of pathogen behaviours capable of generating a first-contact-like pattern are discussed further in the Results and Discussion sections.

When we tested the combinations of maximum pathogen mortality rates (*θ*_1−3_) which could give rise to a first-contact-like pattern in the absence of any protective alleles, we found that such combinations did exist (Fig. 3*a*). The pathogen for which *R*_0_ = 1·5 had to have a mortality rate >0·45, the pathogen for which *R*_0_ = 3·5 could have a mortality rate between 0 and 0·15 and the pathogen for which *R*_0_ = 15 had to have a mortality rate <0·08. It is therefore conceivable that epidemiological and ecological effects alone can account for a first-contact-like pattern.

### If herd immunity affects pathogen mortality rate, first-contact-like patterns can be achieved under a wider range of mortality scenarios, still in the absence of selective processes

Our model also includes parameter *γ*, which, when it takes a non-zero value, makes each pathogen's mortality rate a negative function of the proportion of the population immune to that pathogen. In the absence of protective alleles, increasing the value of *γ* allows a first contact-like pattern to occur under an increasingly wide range of combinations of maximum pathogen mortality rates (*θ*_1−3_), as illustrated in Figure 3(*b, c*).

Marker (1) identifies a region where the pathogen for which *R*_0_ = 3·5 has a high maximum mortality rate (~30%); the pathogen for which *R*_0_ = 15 has a lower maximum mortality rate (<10%), and the pathogen for which *R*_0_ = 1·5 can take a wide range of maximum mortality rates. Such a combination of maximum pathogen mortalities cannot generate a Pacific Island population-like pattern in the absence of herd immunity feedbacks, but is able to when *γ*⩾0·9 [Fig. 3(*b, c*)].

Marker (2) in Figure 3*c* identifies a region where the pathogen for which *R*_0_ = 15 has a high maximum mortality rate (~23%) and the other two pathogens have lower maximum mortality rates (<20%). This mortality scenario is only capable of generating a Pacific Island population-like pattern when the herd immunity feedback is very strong (*γ* = 1).

### Darwinian evolution can only contribute to first-contact-like dynamics if protective alleles start at specific, high, frequencies

Three separate loci independently determine susceptibility to death from infection with the three possible pathogens in our model. For simplicity, we assume that both heterozygotes and homozygotes for a protective allele are completely protected against death from infection with the relevant pathogen. When protective alleles are included, therefore, the maximum infection mortality rates (*θ*_1−3_) apply only to individuals who are homozygous for the wild-type allele at the host locus in question.

**Fig. 4.**
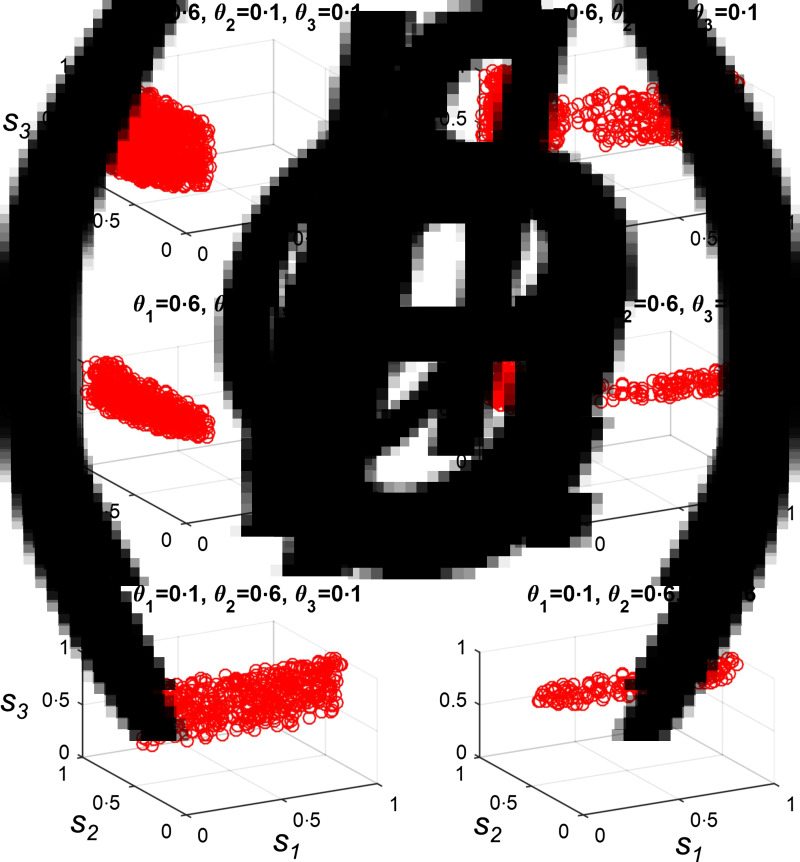
Starting frequencies of protective alleles necessary to create a Pacific Island population first-contact-like pattern if genetic changes can contribute to a drop in mortality rates. We assumed that heterozygosity and homozygosity for each protective allele provided 100% protection against death from the relevant pathogen (i.e. *p*_1*,ijk*_ = 1, where *i* > 1, *p*_2*,ijk*_ = 1, where *j* > 1, etc.). We used Latin Hypercube Sampling to select different starting frequencies for each of the three possible protective alleles, where each allele could start at a frequency between 0 and 1. 25 000 different combinations of starting allele frequencies were tested. Panels (*a*–*f*) illustrate combinations of starting allele frequencies (*s*_1_–*s*_3_) which produced a first-contact-like pattern (as described in the Methods section) under six different pathogen mortality scenarios. *s*_1_ is the starting frequency of the allele that protects against the pathogen where *R*_0_ = 1·5; *s*_2_, *R*_0_ = 3·5 and *s*_3_, *R*_0_ = 15. Values of the three pathogen-specific maximum mortality rates, *θ*_1_, *θ*_2_ and *θ*_3_ are given in the title of each panel. In all panels, *γ* = 0. All other parameters are as given in the Methods section.

We let *θ*_1−3_ take values of 0·1 or 0·6, and tested all permutations of these values, creating eight different pathogen mortality scenarios. We then used Latin Hypercube Sampling to identify combinations of starting frequencies for the three protective alleles which could create first-contact-like dynamics under each scenario. There was no combination of starting allele frequencies which allowed first-contact-like dynamics if neither the pathogen for which *R*_0_ = 1·5 nor the pathogen for which *R*_0_ = 3·5 had a maximum mortality rate of 0·6. However, under all other permutations, first-contact scenarios were possible [Fig. 4(*a*–*f*)]. Allowing the frequencies of protective alleles to evolve under selection from pathogens does, therefore, extend the range of possible pathogen properties which could be consistent with a Pacific Island population pattern – in a similar way to herd immunity feedbacks. However, as can be seen in Figure 4, specific assumptions must be made about the starting frequencies of particular protective alleles in each case.

In keeping with our previous observation that a Pacific Island population-like outcome is possible in the absence of genetic effects if the pathogen for which *R*_0_ = 1·5 has a very high mortality rate and other pathogens have a low mortality rate, when *θ*_1_ = 0·6 but *θ*_2_ and *θ*_3_ = 0·1, the only restrictions on the starting frequencies of protective alleles are that the allele which protects against the pathogen for which *R*_0_ = 1 must be at a low starting frequency (Fig. 4*a*). Under all other circumstances, more restrictive conditions apply to the possible starting frequencies of protective alleles, especially if the pathogen for which *R*_0_ = 15 has a high mortality rate [*θ*_3_ = 0·6, Fig. 4(*c, d, f*)]. If the pathogen for which *R*_0_ = 15 has a high mortality rate, the starting frequency for the allele which protects against the pathogen for which *R*_0_ = 15 (*s*_3_) must be very high.

## DISCUSSION

The exposure of indigenous Pacific Island populations to the pathogens brought by European explorers resulted in massive loss of life. What data are available (Fig. 1) suggest there were catastrophic early mortality events in which 20–70% of island populations died, but no subsequent events to match that level of infectious disease mortality. We had only one census time series for each island, and even those were limited in their scope, thus we did not have the same level of year-to-year sampling for different islands, or enough data to compare within and between island variation. Unfortunately, therefore, it was not feasible to ascertain spatial heterogeneity within this dataset using a measure such as the spatial stratified heterogeneity *q* statistic.

We explored two potential mechanistic explanations for such mortality transitions: one genetic and one reliant on a herd immunity mortality feedback.

Our model included three hypothetical pathogens, with basic reproductive numbers (*R*_0_) of 1·5, 3·5 and 15. Among the pathogens described in the Introduction, the *R*_0_ of measles is estimated to be between 12·5 and 18 [36]; that of smallpox between 3·5 and 6 [37], and the 1918 strain of influenza <4 [38]. The *R*_0_ of *Shigella dysenteriae*, a likely causative agent of dysentery, is unlikely to be much more than 1·5 [39].

We found that a first-contact-like pattern could be obtained without invoking any genetic changes, provided the pathogen for which *R*_0_ = 1·5 (arguably dysentery-like) had a mortality rate of >40%, and that the other two pathogens (influenza, smallpox or measles-like) had lower mortality rates (<15% for the *R*_0_ = 3·5 pathogen, <8% for the *R*_0_ = 15 pathogen). As illustrated in Figure 2, the pathogen with the lowest *R*_0_ will have the lowest peak number of infections per unit time in any given outbreak, and the peak of infections with that pathogen occurs later than those of pathogens with higher basic reproductive numbers. During the second introduction of all three pathogens, these effects are even more pronounced: the herd immunity that exists at the time of the second introduction has a disproportionate effect on the pathogen with the lowest *R*_0_. Ecological interference between all three pathogens, as described by Rohani *et al.* is also likely to contribute to this phenomenon [40]. If the majority of infectious-disease deaths on Pacific islands were caused by low *R*_0_ pathogens subject to such dynamics, the mortality transition does not necessarily require an explanation beyond epidemiological and ecological effects.

Including genetic effects did increase the sets of circumstances under which Pacific Island population-like patterns could be observed. It became possible to observe Pacific Island population-like patterns when the pathogen for which *R*_0_ = 15 had a very high mortality rate [Fig. 4(*c, d, f*)]. However, such scenarios required specific, already very high, starting frequencies of the allele that protected against the pathogen for which *R*_0_ = 15. A role for Darwinian evolution in the shift in mortalities observed across Pacific islands therefore requires that unknown selective pressures were maintaining the same high frequencies of protective alleles at specific loci on many disparate islands before any of the novel pathogens arrived. We cannot rule this scenario out, but it seems unlikely.

We also explored the possibility that infectious disease mortality rates are affected by the level of herd immunity, by making infection mortality a negative function of the proportion of the population already immune to that pathogen. Such a relationship is a potential proxy for one or both of the following phenomena:

- The higher the average age of those infected, the higher the infection mortality (adults are more likely to die from infection than children).
- The higher the proportion of the population infected at a given point in time, the higher the infection mortality rate.

The former of these effects could be mediated by age, e.g. haemorrhagic smallpox and severe varicella being more likely to occur in adults than in children [33]. As for the latter, there is evidence that for measles, intensive exposure to infection is linked to higher case-fatality rates [41], and it is entirely possible that such a relationship holds true for other infectious agents.

We found that the stronger the herd immunity feedback on infection mortality rates (the higher the value of *γ*), the wider the possible range of maximum mortality rates each pathogen could take, and, crucially, scenarios in which the pathogen with the lowest *R*_0_ (*R*_0_ = 1·5) had a relatively low mortality rate became possible [Fig. 3(*b, c*)].

If we assume that smallpox had the highest case-fatality rate of all the pathogens considered in the Introduction, we might tentatively suggest that the scenario indicated by marker 2 in Figure 3*c* is the most plausible. Here the pathogen for which *R*_0_ = 3·5 (arguably smallpox-like or influenza-like) has a consistently high maximum mortality rate (~30%); the pathogen for which *R*_0_ = 15 (arguably measles-like) must have a maximum mortality rate around 10%, and the pathogen for which *R*_0_ = 1·5 (dysentery like) can take a very wide range of mortality rates: suggesting that mortality from this type of pathogen (i) is not essential to create first contact like dynamics, given the presence of the other two pathogens, but (ii) in no way precludes first-contact-like dynamics.

Genotype certainly does affect human susceptibility to infection, and infectious diseases undoubtedly represent a powerful selective force. However, our best-studied examples of human disease resistance alleles under selection from pathogens – the haemoglobin mutations which provide malaria protection – attained their present day frequencies over thousands of years [42, 43]. It has been suggested that natural selection could have accounted for the known decline in tuberculosis mortality between 1830 and 1950, but Lipsitch & Sousa have shown that it is implausible that genetic factors could have brought about such changes in the time available [44]. Galvani & Slatkin considered the possibility that either plague or smallpox may been responsible for increased frequencies of a specific deletion in the CC-chemokine receptor type 5 gene (*CCR5*-Δ*32*) observed in European populations: the smallpox-like pathogen could increase the frequency of *CCR5*-Δ*32* to a frequency of 10%, but this required sustained selection over 680 years [45]. The timescale of the Pacific Island population mortality transition is shorter than the timescales considered by either Lipsitch & Sousa or Galvani & Slatkin, and the models we present here demonstrate that the circumstances under which a genetic mechanism could account for a first-contact-like pattern are restrictive. A herd immunity-linked mechanism seems more likely and is consistent with a wide range of possible pathogen mortality scenarios. If the herd immunity hypothesis is correct, then current Pacific Island populations are at no higher risk of mass mortality from newly emerging infectious diseases than other geographical groups.
